# The science behind soft skills: Do’s and Don’ts for early career researchers and beyond. A review paper from the EU-CardioRNA COST Action CA17129

**DOI:** 10.12688/openreseurope.15746.2

**Published:** 2023-10-12

**Authors:** Shubhra Acharya, Mihai Bogdan Preda, Ioanna Papatheodorou, Dimitra Palioura, Panagiota Giardoglou, Vasiliki Tsata, Sanja Erceg, Teodora Barbalata, Soumaya Ben-Aicha, Fabiana Martino, Laura Nicastro, Antigone Lazou, Dimitris Beis, Fabio Martelli, Miron Sopic, Costanza Emanueli, Dimitris Kardassis, Yvan Devaux, EU-CardioRNA COST Action CA17129.

**Affiliations:** 1 Cardiovascular Research Unit, Department of Precision Health, Luxembourg Institute of Health, Strassen, 1445, Luxembourg; 2 Faculty of Science, Technology and Medicine, University of Luxembourg, Esch-sur-Alzette, 4365, Luxembourg; 3 "Nicolae Simionescu” Institute of Cellular Biology and Pathology, Bucharest, 050568, Romania; 4 School of Biology, Faculty of Sciences, Aristotle University of Thessaloniki, Thessaloniki, 54124, Greece; 5 Developmental Biology, Clinical, Experimental Surgery and Translational Research Center, Biomedical Research Foundation of the Academy of Athens, Athens, 11527, Greece; 6 Department of Medical Biochemistry, Faculty of Pharmacy, University of Belgrade, Belgrade, 11000, Serbia; 7 National Heart and Lung Institute, Imperial College London, London, W120NN, UK; 8 Laboratory of Molecular Cardiology, IRCCS Policlinico San Donato, Milan, 20097, Italy; 9 Laboratory of Biochemistry, Medical School, University of Crete, Heraklion, 71003, Greece; 10 Institute of Molecular Biology and Biotechnology, Foundation For Research & Technology Hellas (FORTH), Heraklion, 71003, Greece

**Keywords:** Soft skills, early career researcher, research ethics, publication writing, career opportunities, academia, industry.

## Abstract

Soft skills are the elementary management, personal, and interpersonal abilities that are vital for an individual to be efficient at workplace or in their personal life. Each work place requires different set of soft skills. Thus, in addition to scientific/technical skills that are easier to access within a short time frame, several key soft skills are essential for the success of a researcher in today’s international work environment. In this paper, the trainees and trainers of the EU-CardioRNA COST Action CA17129 training school on soft skills present basic and advanced soft skills for early career researchers. Here, we particularly emphasize on the importance of transferable and presentation skills, ethics, literature reading and reviewing, research protocol and grant writing, networking, and career opportunities for researchers. All these skills are vital but are often overlooked by some scholars. We also provide tips to ace in aforementioned skills that are crucial in a day-to-day life of early and late career researchers in academia and industry.

## Introduction

**Figure 1.**
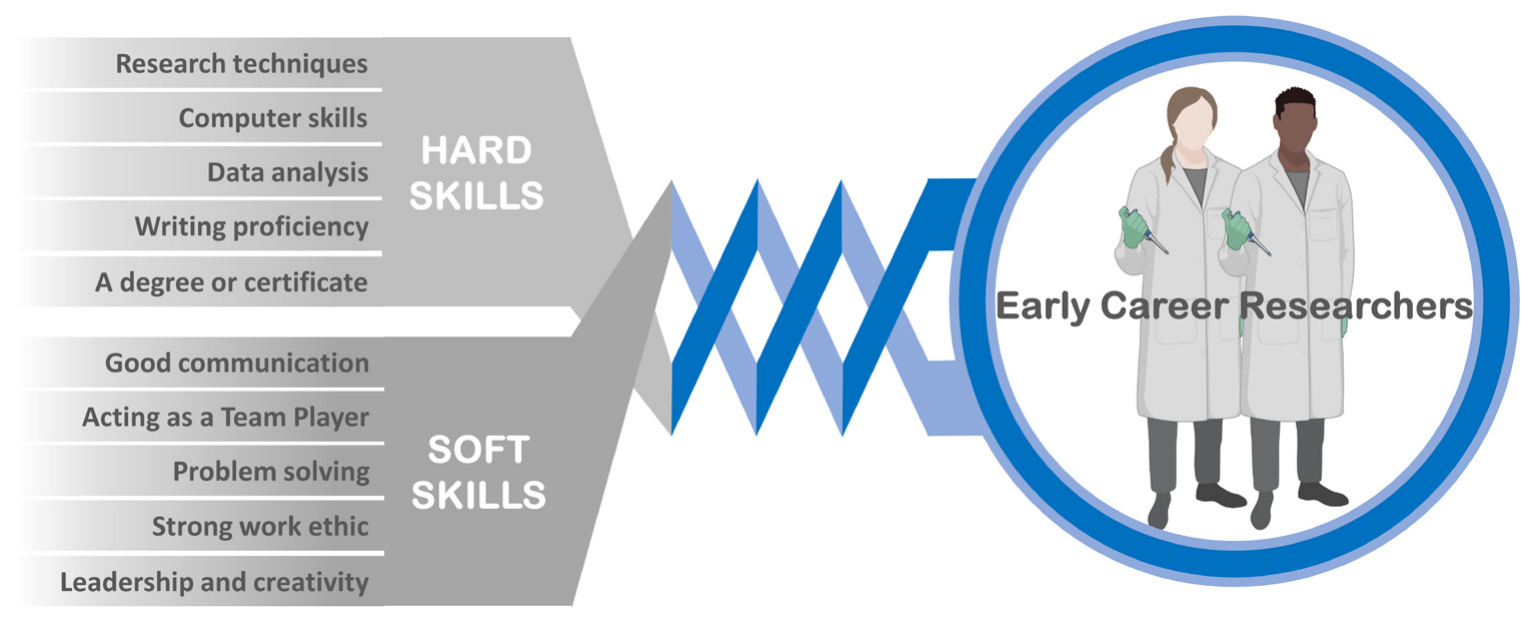
The interrelation of the two distinct sets of “hard” and “soft” skills required for the 21st-century ECR.

The United Nations Educational, Scientific and Cultural Organization (UNESCO), and the Organization for Economic Co-operation and Development (OECD) have recently launched recommendations to help today's students to adapt to the changes, challenges, opportunities, and risks of the twenty-first century digital era ^1, 2^. The investments in the education of the human resource require targeted research and trainings in the essential skills for the new technologies and for the open science concept and practices. One of the first challenges in the Open Science framework is to ensure that the mentors are able to provide all the trainings they think are relevant for the trainees in a sustainable way in order to improve the quality, efficiency, and responsiveness of research. This involves, undoubtedly, the harmonious combination of ‘hard’ and ‘soft’ skills that the early career researchers (ECR) need to acquire in order to thrive in this rapidly changing world ( Figure 1).

‘Hard’ technical skills are easier to learn rapidly but are no longer sufficient for an ECR to withstand in this highly competitive global work environment, and the ‘soft’ social skills, like teamwork and communication, become of paramount importance ^3^. The new generation of ECRs need to be equipped with skills and expertise relevant to workforce participation for decades to come, but unfortunately the unique ‘soft’ skills are currently underexamined in research and undersupplied through education ^4^. Thus, in this position paper, the members of the EU-CardioRNA COST Action CA17129 network ^5^ provide practical guidance about some of the most valuable traits and soft skills young researchers should cultivate to succeed in the labour market.

The views expressed in this article are those of the authors. Publication in Open Research Europe does not imply endorsement of the European Commission.

## Transferable skills

**Figure 2.**
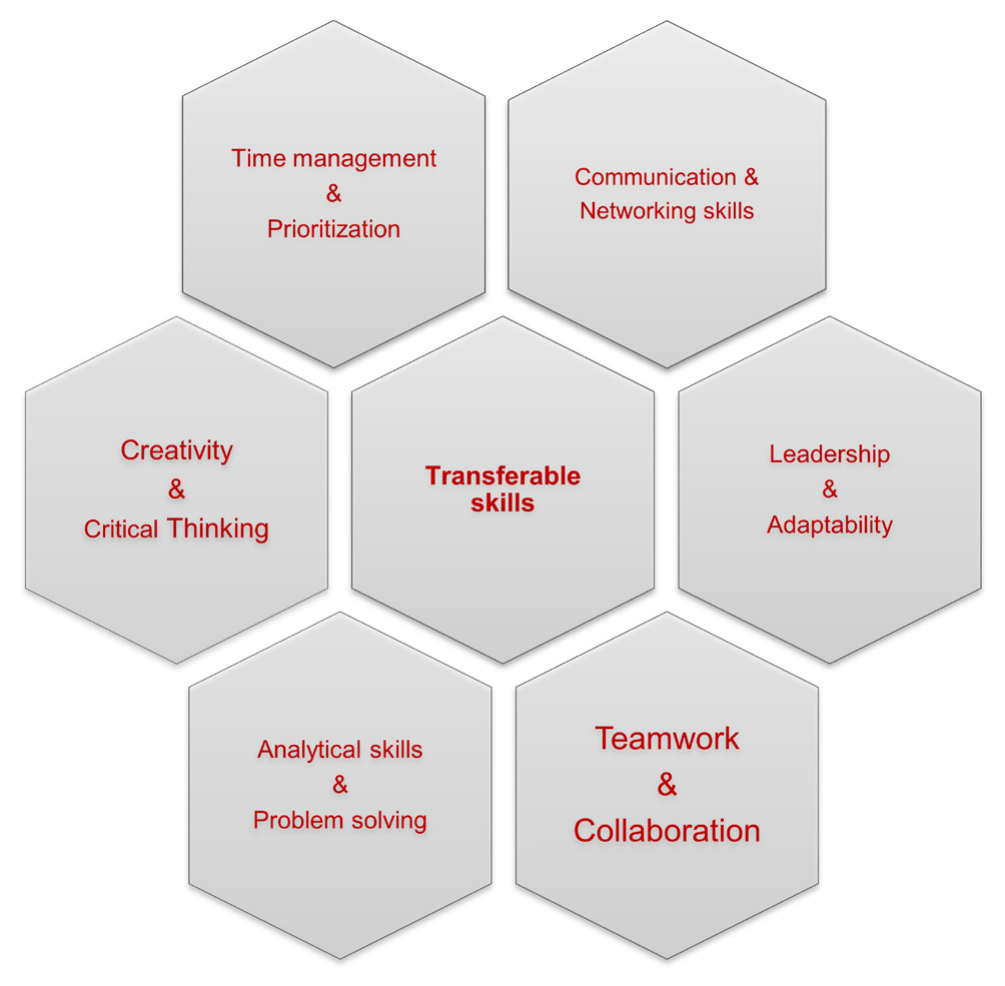
Transferable skills for researchers.

In order to establish and maintain a successful career in research, ECRs need to be equipped with multiple, interdisciplinary assets that can be applied to more than one instance; these skills are named transferable skills. In this first section, we underline the major and most valuable transferable skills, and discuss how young scientists should develop them as part of a successful path in research ( Figure 2). Firstly, since researchers regularly encounter simple or complex problems that require rigorous troubleshooting, they should be able to seek the most appropriate solution based on both their critical thinking and their creativity, in order to deal with the different situations effectively. Critical thinking and effective troubleshooting are also what employers and principal investigators seek for in an ECR. It is also a great asset for employers to incorporate in their groups people that possess analytical skills, meaning people who are able to collect and interpret new information fast and efficiently, analyse it thoroughly and develop effective strategies to approach and salvage any mishap. Intertwined with critical thinking is systems thinking, which involves analyzing complex problems by considering how the different parts of the problem are interconnected and interdependent on each other. By considering all aspects of the problem, systems thinking helps to identify the challenges of implementing and using the innovation. Importantly, working in research requires vigorous multitasking, which consumes time, energy, and great physical and mental labour. Gaining hands-on experience in handling multiple assignments is a smart way in order to manage a more effective allocation of time and it eventually maximizes one’s productivity. However, to avoid mistakes that often appear with multitasking, it is vital to establish an effective time management and organization plan.

Effective time management is a fairly remarkable transferable skill for ECRs. In addition, being able to set realistic goals and completing them in a certain amount of time is vital for meeting the designated deadlines and requires good organizational skills, especially for time-sensitive tasks. Thus, smart prioritization for personal tasks is key but also the time management and overall performance of all the members of a research group should be well-orchestrated to maintain the high-quality work of the team. For that reason, being as productive as one can be in a research group equals to acting as a team player and ECRs need to develop this skill over time. Working in different environments, for example as part of mobility programs, taking on tasks that challenge you to work with different people, that expand your limits, and that get you out of your comfort zone are all very effective ways to learn how to support others in terms of lab work but also to rely on them if need be. It should be noted that teamwork and collaboration skills are usually a prerequisite in job descriptions. Showing true dedication and having a strong sense of responsibility over any collaboration and task are equally important qualities of reliable team members ^6^. However, being persevering, dedicated, and persistent are also intertwined with being able to adapt to any circumstance and overcome any problem, while also maintaining the motivation and momentum to move forward. Thus, the skill to planning/goal setting and forward thinking at the right time of the career is another important aspect for a researcher. The above skills can move an ECR forward and establish a very successful career. Finally, since leading a research group independently is the goal of many ECRs, acquiring strong leadership skills should be an active process of learning from the very early stages in one’s career. Attending leadership courses and taking an active role in training schools or workshops provides young scientists the opportunity to learn and actively practise. Successful leaders are a source of inspiration and motivation for their team members, even under tough situations, while they have the ability to set appropriate goals and foresee problems. Overall, acquiring and developing transferable skills is vital not only during the very first steps of an ECR, but it should be a continuous process throughout one’s career in research.

## Preparing for an interview

**Figure 3.**
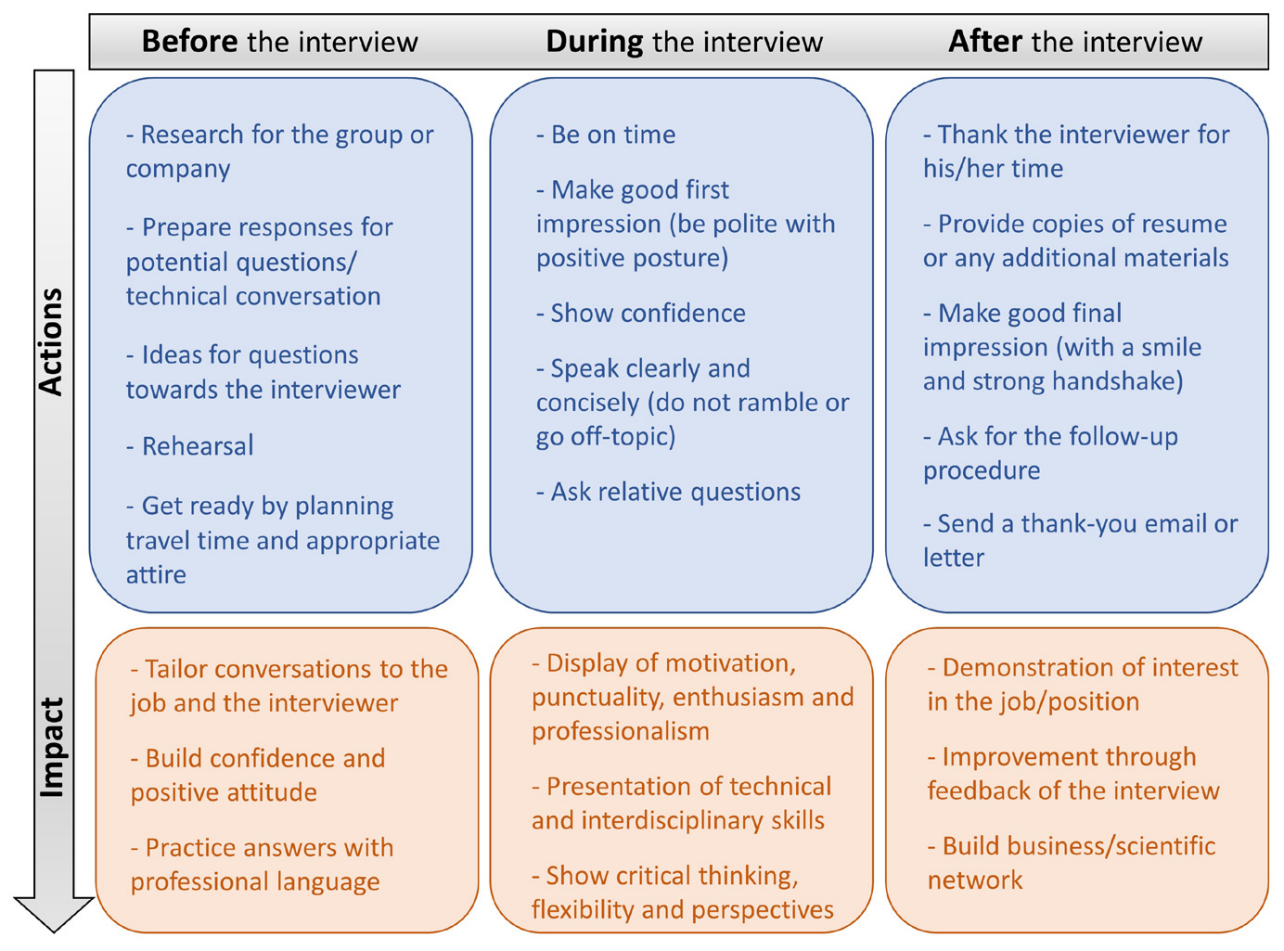
Features for a fruitful interview.

While the first part of obtaining a position is the evaluation of the candidate’s application, interview performance is the next determinant of the decisive assessment of the applicant ^7^. Interviewing is a critical step to successfully obtain a position in both academia and industry. In essence, it is a communication procedure that serves the purpose of exchanging information which will help the employer evaluate the candidate but also concurrently, it will give the opportunity to the employee to access to the critical information on the job position. Therefore, acquiring certain interview skills is essential in order to be effective throughout the selection process. Here, we display critical features for a fruitful interview that can be divided in three instants: before, during, and after the interview ( Figure 3) ^8– 10^.

**Before the interview:** It is highly important to invest time on gathering and reviewing all the information regarding both the job position and the perspective research group or company/team. In case of academia, this could include the main field of scientific interest, detailed list of publications and hosting facilities of the institute/university, whereas in case of industrial position that might include innovations/achievements of the working group and expertise of the specific private sector. Secondly, it is essential for the candidates to be prepared regarding possible conversation topics and how they should address the potential interview questions. On that note, creating a list of previous job experience including challenges and achievements the candidate has faced and rehearsing all the aforementioned issues could strengthen the responses and build the confidence of the interviewee.

**During the interview:** On the day of the interview, punctuality is highly appreciated. The candidate should reach the location of the meeting slightly before the scheduled time considering the required transportation time along with possible unforeseen factors. Most interviewers aim to seek for skills, motivation, enthusiasm, and professionalism on the job candidate. Therefore, attending the interview with an appropriate attire, positive posture and voice-tone will gain a great first impression. Communication skills (verbal and nonverbal) are vital for the success of an interview. During the interview, the candidate must respond to the questions clearly and concisely as well as listen attentively. Undoubtedly, confidence and use of professional language are important components with significant impact on the general interview performance. Finally, the interviewee should have prepared a list of questions to ask regarding the position (research line/project), the work environment, and policy while avoiding inquiries of salary/vacation as first question.

**After the interview:** As the interview is completed, the candidate could thank the interviewer for the chance to attend, show genuinely interest in the position, and kindly ask to be informed about the outcome of the discussion. This could be either the interviewers deciding on a candidate or to be followed by a further step of a second interview. In case of rejection, the interviewee may request feedback in order to improve the interview performance in future cases.

## Good scientific practices- a key to perform ethical research

In the previous chapter, we described a toolbox that could assist ECRs in landing their dream job for the interview. What ensures, though, that the job is done right? From human stem cells and organoid research to animal experimentation and clinical trials, biomedical research involves a wide range of ethical concerns that confront scientists with moral decisions ^11^.

To create a framework in which animal research is performed in humane conditions, Russell and Burch introduced the principles of the 3Rs (Replacement, Reduction, and Refinement), that should already be considered, when designing any experimental protocol involving animals. These aim to i) replace animals used for scientific reasons with other alternatives, ii) reduce their number to the minimum required to obtain reproducible results, and iii) ensure the animals’ welfare throughout its life and the duration of the scientific procedure by minimizing pain and/or suffering ^12^. More recently, the ARRIVE guidelines have come to complement the 3Rs in order to ensure that publications describing animal research are reported in enough detail to add to the knowledge base ^13^. In this regard, the United Nations has proposed Sustainable Development Goals (SDGs; https://sdgs.un.org/goals). Following the SDGs, it is essential for the researchers to focus on both digital and green skills which could help in the use of technology to manage and solve problems and to reduce the environmental impact of research activities ensuring the sustainability of health systems.

Similarly, to reflect and address the complicated ethical issues that arise with scientific and technological advances in the fields of 1) genome editing, 2) embryo culture, embryo models and gametogenesis research, and 3) organoid and chimera research, the International Society for Stem Cell Research regularly updates its guidelines ^14^. These guidelines set up specific standards not only for scientists and clinicians treating patients, but also for policymakers, funders, and potential recipients of any treatments that result from it. They define clear and external boundaries ensuring that biomedical research is conducted and communicated with ethical integrity.

Biomedical research is being transformed through the use of high-throughput genome sequencing of humans using big data approaches that may lead to personalized / precision medicine. While our knowledge of what our genomic data means deepens by the day, lots of variants are still uncharacterized and have unknown significance. Several ethical issues should be addressed when dealing with releasing human genomic data. These include, for example, family members of patients with genetic predisposition to a disease, life insurance policies, and informed consent policies concerning patients ^15, 16^. To ensure proper collection, handling and release of such information, the European Union (EU) has enforced the General Data Protection Regulation (GDPR), a privacy and security law that sets a strict framework onto organizations collecting data related to people in the EU and protects the right to privacy for every individual ^17^. 

It should be noted that ethics is not limited to issues that are generally supervised by ethics committees for human and animal studies. Correctly performed, recorded, and communicated science is also intrinsically ethical ^18^. Effort has been put in recent years to highlight the importance of good data handling, management, and presentation to promote transparency in all scientific studies, irrespective of the field or the sample size. According to the FAIR Principles, data should be findable, accessible, interoperable, and reusable (FAIR), putting specific emphasis on enhancing the ability of machines to automatically find and use acquired data, in addition to supporting its reuse by individuals ^19^. Focus should be also given during data reporting and especially on choosing the correct type of graphic (i.e., dot, box, and/or violin plot) to accurately present the data depending on the study design, the sample size, and the type of variable, allowing a direct evaluation of individual data points, their distribution, and their statistical analysis to display the data as they are 20. Another more technical aspect to be taken into account is image data processing as image manipulation can in some cases be classified as scientific misconduct. In cell biology, images often serve as primary data and for the microscopy field “seeing is believing” as stated by Prof. Alison North in her publication ^21^. However, digital manipulation can be done very easily nowadays and it can be difficult to find the ethical lines of what is and what is not allowed in digital manipulation of the scientific raw data. Imaging processing workflows have been introduced that allow authors to present images effectively and ethically while publishing truthful and legible images ^22, 23^. Images and their containing elements should be properly annotated and scaled, while colors should be chosen wisely, allowing people with color vision deficiencies to efficiently distinguish them (for example: red–green color blindness is the most common form of color vision deficiency) ^24^. Furthermore, image manipulations such as i) non-linear color, brightness, and contrast adjustments, ii) cropping, re-sizing, and selective enhancements of specific parts, and iii) cloning of objects in an image in which they did not previously exist are considered a misconduct and should be avoided or clearly stated ^25, 26^.

Finally, in an era of immense scientific output, researchers -but also publishing groups- should be actively trained in original writing, avoiding plagiarism -in all of its forms 27, building a strong, ethical science culture and keeping literature and academia honest.

## Scientific communication - Oral and presentation skills

Along with performing good research, ECRs must know how to disseminate their research to benefit a wider audience. Scientific conferences are one of most common ways for ECRs to communicate their work and to gain wider exposure. These are events that bring together researchers of similar interests, encouraging them to discuss and exchange views on topics from specific areas. The oral presentations represent the main focus of conferences, but the ones that provide the most opportunities for interaction, and therefore networking, are posters. The oral presentations are mostly reserved for more experienced researchers, while posters are attended mainly by younger researchers. Both of them have their own advantages ^28^. Thus, for maximizing an ECR’s exposure to conferences, there are several written and unwritten rules that could be followed.

1. Selection of appropriate conferences - To meet learning and networking needs, it is important to choose the appropriate conference, whether it is local, national, or international ^28^. The conference topic should be evaluated first as well as potential conference participants ^28– 30^. Once the conference has been selected, the next step would be submitting an abstract. If the abstract is accepted, it will be included in the conference proceedings that allow conference participants to read it beforehand. Only selected abstracts will be designated for oral presentations, while the rest will be transformed into poster presentations ^28^.
2. Know your stakeholders- Special attention should be paid to whom a researcher will present their work ^28– 30^. It is not the same if there are researchers of similar interests in the audience or if there are people with only marginal interests. For example, if scientists in a related field are addressed, then the focus will be on the methodology part, and if there are clinicians in the audience, the focus will be on connecting a particular discovery with a diagnosis, prevention, and treatment of a disease. So, it should be kept in mind what are the expectations of the audience and how the researcher’s presentation can bring them benefits ^29^.
3. Keep calm- Nervousness is always present before and during a presentation of any kind. The way to fight it is to be well-prepared. As a part of the preparation, a speaker should practice in front of their colleagues, who are familiar enough with the work and thus can make useful corrections. The anxiety is on the highest level at the beginning of a presentation, and as it progresses, a speaker becomes more relaxed, taking care mostly of the remaining time ^29^. During lectures, notes may be useful, but they should be in some form of bullet points, not long paragraphs ^28, 30^.
4. Time limits- Whether it is an oral or a poster presentation, speakers must consider the time they have available ^29, 30^. Oral presentations usually last 10–20 minutes, sometimes up to an hour. Occasionally the questions at the end are counted within the given time, so it is important to consider those as well. It is advisable to prepare a complete presentation within the duration of 80 percent of the given time. At the event, the presentation time is extended mainly due to the numerous pauses that are unconsciously taken while waiting for the reaction of the audience ^29^. Exceeding the available time is considered very unprofessional because it disrupts the busy schedule of the conference, and also distracts the audience who needs a lot of concentration to follow all the sessions during the day ^29, 30^. Posters are also time-limited. In moderated poster sessions, each presenter has three minutes on average to explain the basic concept of their work and then answer the delegates’ questions. There is definitely not enough time for the presenter to explain the complete research, as well as for the viewers to absolutely understand the topic. The emphasis should be on opening the door for possible cooperation in the future ^28^.
5. Make it interactive- Both oral and poster presentations must be accompanied by appropriate visual material. It allows the speaker to follow the story, and the audience to concentrate, because listening without accompanying visual material is quite challenging. If the necessary equipment is available and functional, and visual material is prepared in advance, the presenter does not have to think about it during their speech ^28, 29^. Table 1 presents the general rules for presentation design, both for oral and poster presentations, as well as what should be avoided ^28– 30^.
6. Dress code- Although scientific conferences do not require participants to follow a specific dress code, there are some unwritten rules that should be kept in mind when it comes to dressing. It is important to be relaxed enough in the clothes that are worn, but more formal is better than less formal. In that manner, the audience feels respected. The way the presenter is dressed up can greatly affect their professional image ^29^.

**Table 1.** General rules for presentation design for early career researchers.

		Do's	Don'ts
General rules	**Font/** **background**	-Sans serif (Arial, Helvetica, Franklin Gothe) -Caps in headings -Appropriate size -Font/Background contrast	-Serif (Times New Roman, Georgia, Rockwell) -Caps in paragraphs -12 pt cannot be read even in the first row -Dark colored background
	**Figures**	-Strongly recommended -High quality -Self-designed or cited	-Blurred and low-quality -Plagiarism -Overcomplicated
	**Tables**	-Only if necessary -Easy to follow	-Too many tables -Do not emphasize the point
	**Graphs**	-Choose the right type -The data can be followed by non-expert -Legends are not necessary -Name the graph properly -Proper scaling	-Overloaded graphs -Too many decimals and unrecognizable symbols -Multiple graphs indifferent styles
Specific rules	**Presentation**	-Number of slides: one minute per slide -Visible to everyone -The research topic, name, and surname of the presenter and date must be stated at the beginning -two-three sentences on each slide (bullet points)	-Long illegible paragraphs -Take-home messages not included
	**Poster**	-Follow dimensions defined by the congress organizer -Use maximum dimensions allowed, for visibility and prominence -Title and general layout should be visible from three meters -A balance between text and figures -QR code could be included	-The dimension and orientation of the poster ─ portrait or landscape ─ were not taken into account -Too much text = a newspaper article -Too many figures = unscientific poster

On the other hand, there are things that cannot be predicted and influenced, but it is important to be aware of them. It may happen that the projector does not work for some reason, that the microphone is turned off suddenly, that the laser pointer does not work because the battery is low, and many more. In these cases, the speaker should remain calm and relaxed enough to find an alternative solution, for example, to speak louder or to point directly at the screen ^29^. Being confident and well prepared is the key to a good presentation.

## Literature reading and reviewing articles

### Literature reading

Literature reviewing is a necessary and important activity in the academic life of an ECR. It is very likely one will need to include a literature review during their research career either for a long report, a dissertation, or a PhD thesis. However, the flood of scientific papers available today might paradoxically prevent finding relevant literature and stop new ideas from appearing. There is a high burden to find and choose right papers and learn about a particular field ^31^. For this concern, choosing journals that publish ‘good’ science, meaning systematic, rigorous, and reproducible research is imperative. On the other hand, one needs to be open-minded and not only track the credentials linked to reputable scientific journals and top scientists.

In this section, we focus on the difficulties that an ECR may experience in following the literature and offer instructions to perform this exciting scientific activity. Firstly, a comprehensive understanding of a scientific paper requires more than one reading, making it an effort of several hours. Generally, one should start reading the title and the abstract, followed by the conclusions. This helps in understanding if the goal of the described work is of interest for one’s own study. In the first comprehensive reading, the focus should be on getting general overview of the aims and methods. Then, the focus must be directed on the details of the methodology, results, and interpretation. Following, we list some tips for critical assessment of a scientific article:

1. Keep an open mind to the findings outlined in the article.
2. Read and summarize each article noting its main findings and impressions.
3. Examine each article for the strengths and weaknesses related to credibility and authenticity or appropriate standards.
4. Try to extract the unique central ideas of the article.
5. Look for points of difference between articles.

### Literature reviewing

**Figure 4.**
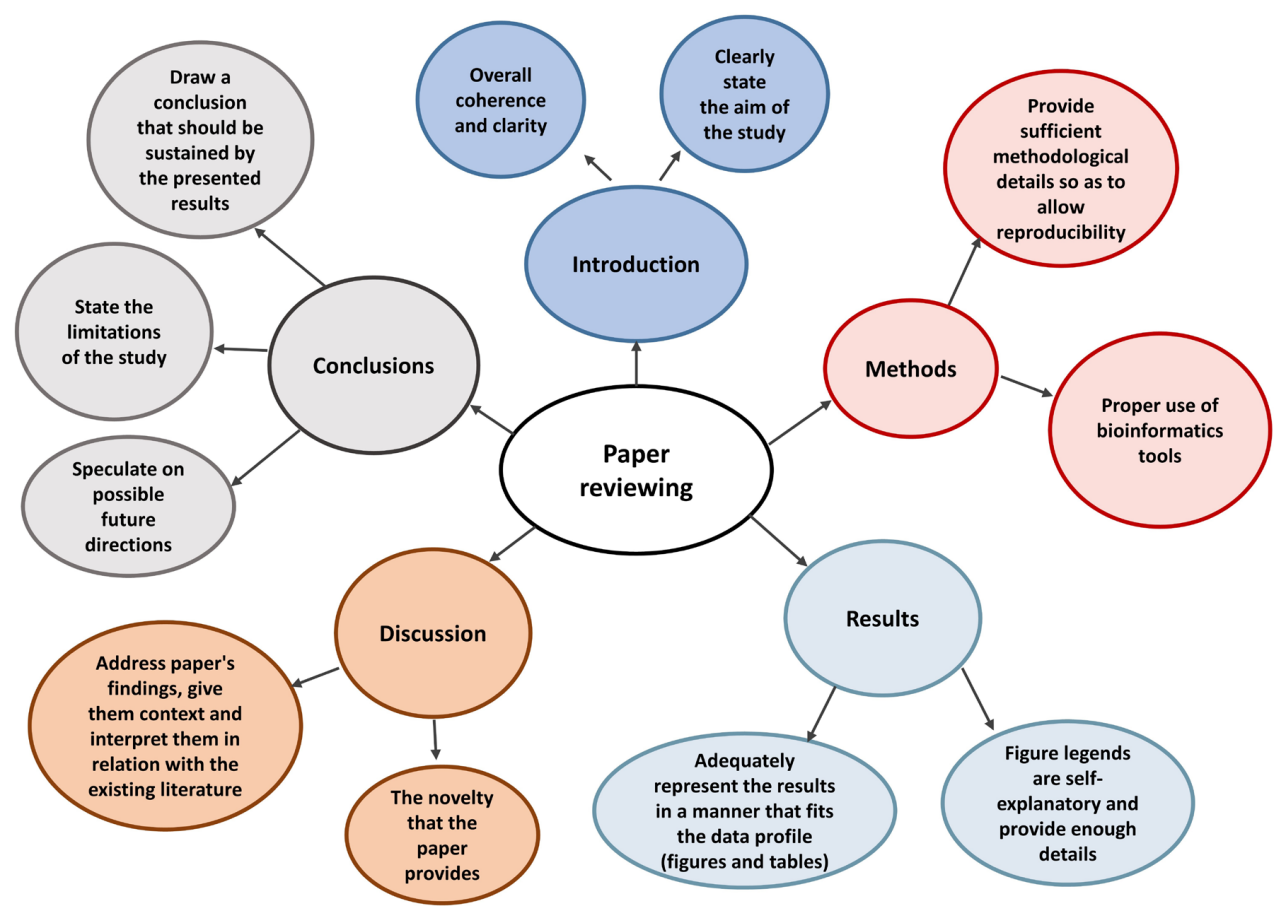
Components of a paper reviewing process.

With the advancement of career and increased experience in the field, different journals might contact the researcher to serve as a peer reviewer for a potential article. This can be important as it shows that other researchers in the field recognize one’s expertise. a, such as MDPI, Elsevier, or Wiley ^32– 34^. Unfortunately, the skill of reviewing a paper is something that is rarely taught during one’s graduate degree program, so many ECRs are left feeling ill equipped in this area ^35^. In this section, we discuss the main aspects that should be considered when reviewing a scientific paper. Since the peer-review method of a potential scientific paper is the way to ensure that the respective paper is accurate and in its full potential, its importance cannot be stressed enough ^36^. Following points must be taken in account while reviewing a paper:

1. Does the paper fall within the scope of the journal or the special issue? The editor, who also checks if the manuscript follows the structure guidelines of the journal, usually takes this decision. After editors decision, the manuscript is forwarded to reviewers, who have expertise in the respective research field ^35^.
2. The overall coherence of paper must be taken into account. The aim of the study and how well the obtained results demonstrate it must be clearly mentioned.
3. The abstract must accurately summarize the main aim, methods and findings of the study. A graphical abstract could be used to visually describe the same.
4. The methods section should have sufficient details for other researchers to reproduce the experiments if needed. Another aspect regarding methodology is data processing; a clear description of informatics tools can highly enhance the quality of a paper.
5. In the results section, authors should not re-mention the details already found in tables or figures, rather highlight the trend and significance of these data. The figures and graphs in the manuscript, with appropriate title and legend, are the ones that tell the story of the paper and attract the reader. Thus, adequate representation of the results to fit the data profile is extremely important. The figure legends should be self-explanatory, containing a suggestive title, the sample size, methodology used, and the full terms alongside any abbreviations.
6. The discussion section of the paper should address its findings, give them context and interpret them in relation with the existing literature, making sure that the references used fit the idea emitted by the respective paragraph. The authors should try to cite other researchers’ work before and then their own previous work to a reasonable extent. In addition, the novelty that the paper brings to the field and if it furthers current knowledge, along with study limitations should be precisely mentioned in the discussion. It must be noted that novelty might be a critical criteria for some journals, while for others it might not be as important.
7. In the conclusions section of the paper, authors should draw an inference that should be sustained by the presented results (exaggeration of the importance of the results is not advised) and speculate (but not over speculate) on possible future directions of their research work. All these steps to be followed during the review process are summarized in Figure 4.

## Research protocol and grant writing

Reading and writing are basic skills that are acquired throughout the education of a young person. Young researchers who have decided to pursue an academic path will need to develop a set of additional scientific writing skills in order to support their research activity. The three most important types of scientific writing skills are research protocol writing, scientific publication writing, and grant writing.

### Writing a research protocol

In the early stages of an ECR’s career, research protocol writing is often done primarily by the supervisor or other experienced researchers from the laboratory, and so the ECR does not have to worry too much about this issue. However, from the very beginning, all ECRs must learn and practice their scientific writing skills in order to become successful scientists.

**Figure 5.**
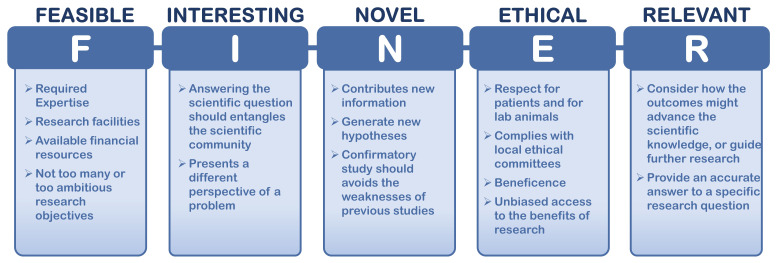
FINER criteria for a good research protocol. Adapted from 41.

Writing a research protocol could be a difficult and time-consuming process and it is important to emphasize that protocols have different features when it comes to clinical trials or basic and translational research. A protocol for a randomized clinical trial is a framework of a clinical study, demonstrating the guidelines for conducting the trial. Any clinical trial protocol must be registered and must conform the international standard for trials protocols (SPIRIT guidelines) ^37^. This topic has been well described in several review articles ^38– 40^ and it will not be our focus in this chapter. Another comprehensive description of clinical trial protocols can be found in the book by Hulley *et al.,* ‘Designing Clinical Research, 4th Edition’ ^41^. In their book, Hulley *et al.,* developed the FINER (feasible, interesting, novel, ethical, relevant) criteria that helps researchers formulate a solid research question, by highlighting useful concepts ( Figure 5). The FINER criteria is particularly useful for ECRs as they can be guided on how to find a good research question and how to design an efficient research protocol.

In basic or in translational research, a research protocol is a detailed and well-structured plan of a project. It can be also an essential component of a research proposal submitted for funding. The plan is structured in a document that specifies the systematic details of a research study, starting from the hypothesis, rationale and background information, primary and secondary objectives, approach or methodology, data management, ethical and gender issues, and statistical analysis.

The scientific process is an activity that involves the rejection of hypotheses that are inconsistent with the experimental results. Testing a hypothesis is what we call an experiment and for a hypothesis to be not only valid but also valuable, a good and detailed research protocol is required. When writing a research protocol, following points should be addressed:

1. The project title should be as clear as possible. It should not be lengthy and should be accompanied by an acronym to be used throughout the text;
2. The project summary should define all the research objectives and the rationale so that there are no doubts that the proposed research is timely and addresses a scientifically important subject;
3. Schemes often help reviewers to understand the strategic plan;
4. The methodology should be described and justified appropriately;
5. Ethical considerations are very critical especially when dealing with humans or experimental animals; risk management and contingency plan must not be forgotten.

The most important element for a successful research proposal is the original hypothesis (or idea). Where do good research ideas come from? It is imperative for an ECR to continuously read relevant literature, actively attend meetings, discuss with colleagues, and to go back to previous data to develop new ideas.

### Writing a grant

A research protocol can be a main part of a grant proposal that is submitted to funding agencies. Grants are the primary source of funding and, consequently, the engine that allows academic research to exist. Grant applications need to be carefully prepared and written in advance to allow several rounds of proof-reading and increase the chances of being financially supported. For ECRs, writing a proposal for a research grant or for a fellowship can be the first step towards scientific independence.

A grant proposal is an unambiguous, direct document written to a particular organization or funding agency to persuade the reviewers to provide you with financial support because: 1) you have a clear idea with a valuable aim that tackles an important and timely matter, and 2) you are capable of implementing that plan.

**Figure 6.**
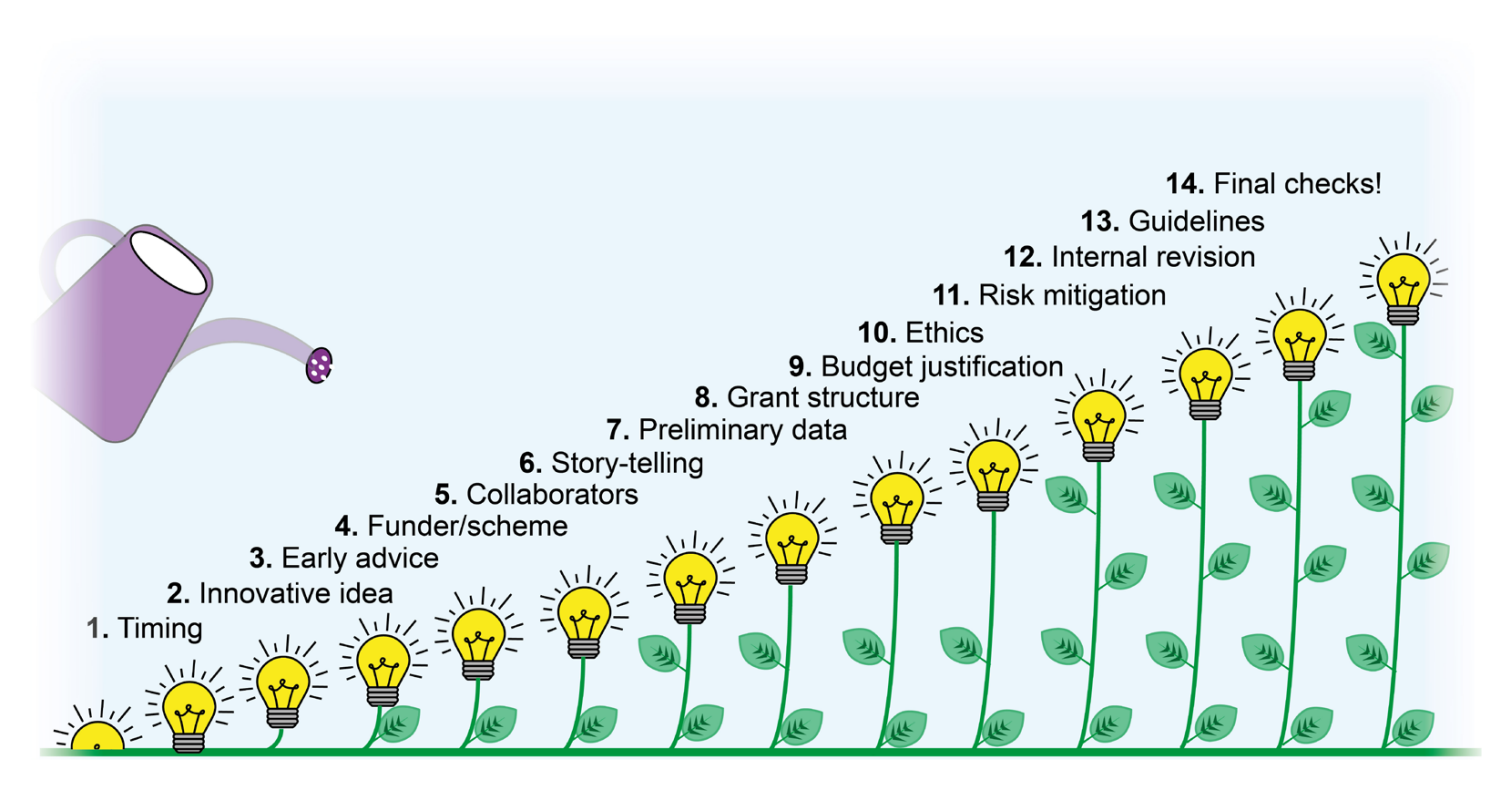
Tips to writing a successful grant application.

To succeed, it is vital to build grant-writing skills ^42, 43^. There are a few steps to follow in order to write a successful grant application ( Figure 6), including:

1. Get the timing right. While scheduling the writing time, it is important to allow enough time for rewrites, proofreads, and unforeseeable events.
2. Formulate an impactful scientific question. Having a clear scientific hypothesis is essential to succeed. The proposed idea must be novel, timely, and increasingly important to funding bodies.
3. Get advice at an early stage. This allows increasing the chances of success and formulating a clear, ambitious but realistic objective. It is recommended to seek suggestions from a range of sources.
4. Choose the right funder and scheme for your proposal. Doing extensive research of available grants and identifying different research interests, missions and priorities of funding bodies will help to increase the likelihood of funding success.
5. Get the right partners. Demonstrating having the right background to carry on the proposed project is essential. Collaborating with experts in the different research areas covered by the project will help counteract criticism during the reviewing process and expand your network.
6. Tell a compelling story with clear language. The project needs to deliver your message clearly and concisely. Obey the three Cs rule: Concise - Clear - Complete ^44^.
7. Include relevant preliminary data. Showing solid preliminary data will support the credibility and feasibility of your scientific hypothesis and the proposed methodology, thus helping convince the evaluation panel.
8. A good grant structure is key. Divide your proposal into stand-alone but also interconnected sections (work packages). Use schemes to convey your message.
9. Justify your budget. Convince the reviewers that the proposed personnel and consumables will be sufficient for the described project and that no shortage of resources will be faced. On the other side, an inefficient use of the resources is equally negative, decreasing the chances to be funded.
10. Consider all ethics issues. If your project involves human subjects or animals, make sure that you have done your ethics self-assessment before submitting the proposal and familiarize yourself with all national or international regulations.
11. Mitigate the risks. Each proposal is exposed to criticism, and each study has limitations. Presenting a risk mitigation strategy within your proposal will increase the credibility of your project and yours as a research leader.
12. Get your proposal reviewed internally. Asking other people to read your proposal will improve its clarity, structure and accessibility.
13. Follow the guidelines. Each grant application comes with specific guidelines. Usually, a template is also provided. Make sure you read the guidelines before you start writing and follow them strictly.
14. Never give up on the final checks. Check and double-check punctuation, presentation and grammar. This will determine how people will perceive your work.

Despite all the points mentioned above, there is no guarantee that a grant proposal will be funded. Getting a grant is very difficult. Competition is usually stiff. However, the process of writing your project in a well-structured proposal helps to improve the hypothesis, the impact and the approach regardless of the final evaluation committee decision. Also, paying attention to reviewers’ feedback after a rejection helps strengthen future proposals, thus increasing the chances of success.

Considering the best-case scenario, the grant gets funded! However, the writing will not be over. In fact, many grants require progress reports and updates, so be prepared to keep on writing and developing your grant writing skills.

### Writing a scientific publication

If funds are secured, and research work is preformed rigorously, the time comes to share the data obtained and make the research accessible for the scientific community. In order to do that it is essential to present and contextualize the results in an understandable way, following some general criteria and particular requirements (author guidelines) imposed by the chosen journal.

Choosing the right journal for your manuscript is extremely important, as it will determine the target audience and the impact of your research. Journal selection is a daunting task. It requires a careful evaluation of the aim and scope of the journal, the peer-reviewing process, the journal indexing, its network and the publishing time. Once the authors agree on the journal, the main author has to download the journal guidelines and prepare the first draft accordingly. Presenting the data in an organized and clear manner is a must. Exposing the draft to several rounds of revisions by all authors will help to improve its quality. Additionally, if important gaps are detected, further scientific work should be performed before the submission to avoid an editorial rejection. Opposite to grant writing, no strict deadline is set, but writing needs to be planned in advance in order to be efficient and not to compromise the novelty of the work performed. Once all authors are in accordance with the good quality of the research and the writing, your paper is ready for submission. In the best scenario, after passing the editorial selection, the quality of the manuscript will be assessed by peer reviewers and revisions will be requested. Regardless of the type of revisions requested (major or minor), be prepared to work hard and fast to answer the reviewers’ questions. Despite being often a stressful process, the aim of peer reviewing is to improve the quality and impact of your work and make your research more clear and accessible for the expert scientific community.

## Networking as a key to success in research

**Figure 7.**
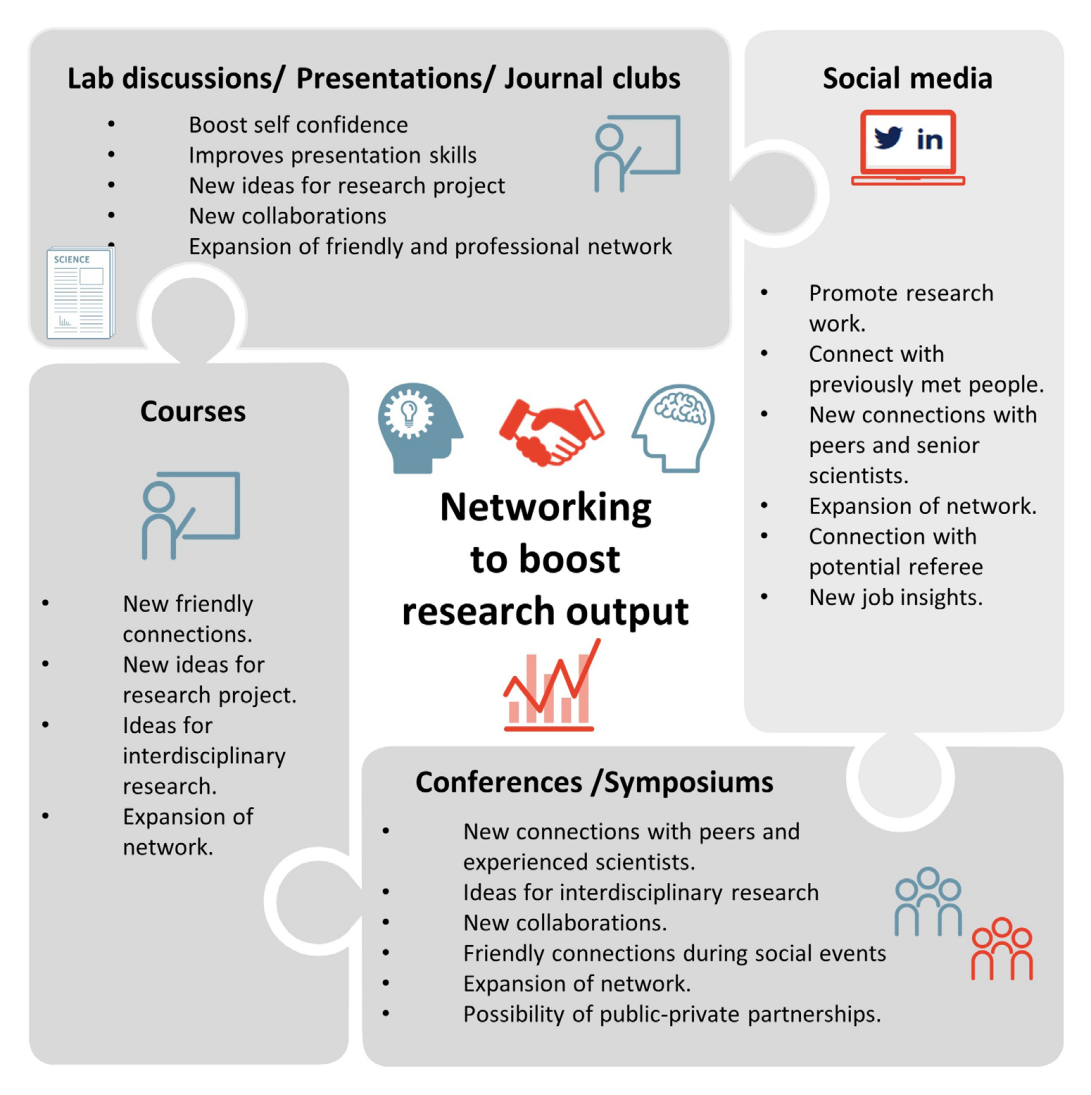
Different networking activities for researchers and expected outcomes.

Networking is part of our day-to-day lives. It helps in establishing and building new relationships in both personal and professional aspects. In this chapter, we discuss how networking can help in technical advancements, technology transfer, collaborations, career development and professional success in research ^45^. It should be noted that research performed in an isolated atmosphere, is less efficient than performed in collaboration. Thus, it is imperative to know the type of networking activities researchers can and should engage into and the outputs to expect from these activities. Figure 7 shows a puzzle of networking activities and their expected outcomes.

### Networking activities

Attendance in networking activities can help to improve communication skills and to boost career opportunities ^46^. For ECRs, the most recommended way to initiate communication is by being regularly active in scientific/non-scientific discussions and presenting their research work in laboratory or in institutional meetings. Initiating a conversation and presenting one’s opinion to experienced scientists and professors might get overwhelming for some researchers. Thus, to widen the network, researchers can start with attending courses at their university or abroad- to expand the network with their peers which indirectly boosts the confidence. Further, attending scientific conferences and presenting their work to establish network with peers along with experienced scientists helps build important connections that significantly enhance the research capacities and boost research outputs. Coffee breaks and social events like dinners or excursions organised during the conferences could help in making professional as well as friendly connections ^47^. The researcher must contact and connect with peers and senior scientists via email or appropriate social media tools after the meeting to stay in touch with them. The use of social media has become imperative in these times. Posting about your lab activities, new publications or simply commenting your opinion on other researchers’ post might significantly help in promoting the research work and broadening the network ^48^. Taking initiative and volunteering to co-ordinate meetings is another way for ECRs to increase their network and confidence. Activities like taking part in journal clubs is also a great opportunity to widen the research horizon. In this regards, ECRs from the EU-CardioRNA COST Action CA17129 network ^5^ have initiated a monthly journal club that aims to catalyse scientific discussions and collaborations between ECRs and senior scientists from across the globe.

### Networking outputs

Each type of networking activity will produce different outputs. To start with, regular communication and liaising with peers improves a researcher’s confidence, which is important to communicate with senior scientists. Attending courses in the university and abroad also helps a researcher to widen their perspective and get ideas to conduct inter-disciplinary research. Other activities like conferences and symposia can help in building connections that can lead to collaborations for ongoing and future projects ^49^. The established connection with a person could also turn into a potential referee for future employments. Another important outcome that needs to be highlighted is successful public-private partnerships. Private companies often sponsor international conferences where they have representatives who present their recent research developments. Collaborations with these private companies can broaden the research outcomes to a translational level. This creates a high impact not only to the researcher’s personal profile but also makes a synergistic effect in improving the need of novel therapies for improved healthcare ^50^.

One important aspect when it comes to participating in networking activities is funding. Several government and private agencies financially support these networking activities, helping researchers, especially ECRs, to attend scientific meetings with the final goal to boost their research outcome. One such example is the European COST Association (European Cooperation in Science and Technology; www.cost.eu) funded by the Horizon Europe research and innovation framework programme. COST supports the so-called “Actions” which are networks of scientists and key stakeholders aiming to engage into networking activities (meetings, short-term scientific missions, conference grants and so on) towards the satisfaction of an innovative and unmet research topic, while contributing to catalyse collaborations between scientists and boost the career of young researchers. The European Union's flagship programme ‘Marie Skłodowska-Curie Actions’ is another example of funding schemes aiming to support scientific excellence and cooperation across countries, sectors and research fields, particularly adapted for ECRs ^51^. Likewise, several similar funding bodies in different countries support grants for networking to promote transfer of technology for inter-disciplinary research and to promote inter-institutional and public-private collaborations.

## Career development in academic and/or private sector

The ‘publish or perish’ philosophy together with the constant technological advancements puts a lot of pressure on the ECR community. Keeping up the pace in order to build up a successful career in the academic sector seems very challenging ^52^. Thus, it is of great importance for an ECR to carefully plan their roadmap to a successful academic career. During the course of the Ph.D. thesis, researchers should acquire all the technical skills that are state-of-the-art in the field, and improve critical thinking, problem-solving, and scientific writing skills. Equally important, during this period researchers should start to build international liaisons that will be exploited in the later stages of their careers for post-doctoral studies, collaborations, and international projects. With the ever-growing need for an interdisciplinary approach in research, it is essential to establish good connections with reliable and competent peers and work together on the realization of new ideas and concepts ^53^. This is even more relevant for researchers coming from developing countries ^54^. After finishing a Ph.D., the researcher should have a clear idea about the next career steps. The researcher interested in a traditional academic career (tenure positions at faculty, research positions at institutes), should proceed with a post-doctoral program, preferably outside the university where the Ph.D. thesis was obtained. By going abroad, the researcher gains more international recognition, expands their cultural horizon, and acquires social skills, thus gaining qualities that will be useful for becoming an independent researcher and eventually a team leader. The criteria for the choice of post-doctoral position should not only be related to the level of excellence of the research facilities, but should also include an assessment of the working environment and if it is stimulating and encouraging enough for the development of the independent researcher ^55^. Considering that after a post-doctoral period, the researcher should be a fully competent group leader, in addition to research skills, it is important to take time during the post-doctoral period to master grant writing skills, management, and leadership skills. By fostering good research practice, international collaborations, and project leading, there is no doubt that the researcher will have a successful academic career.

**Figure 8.**
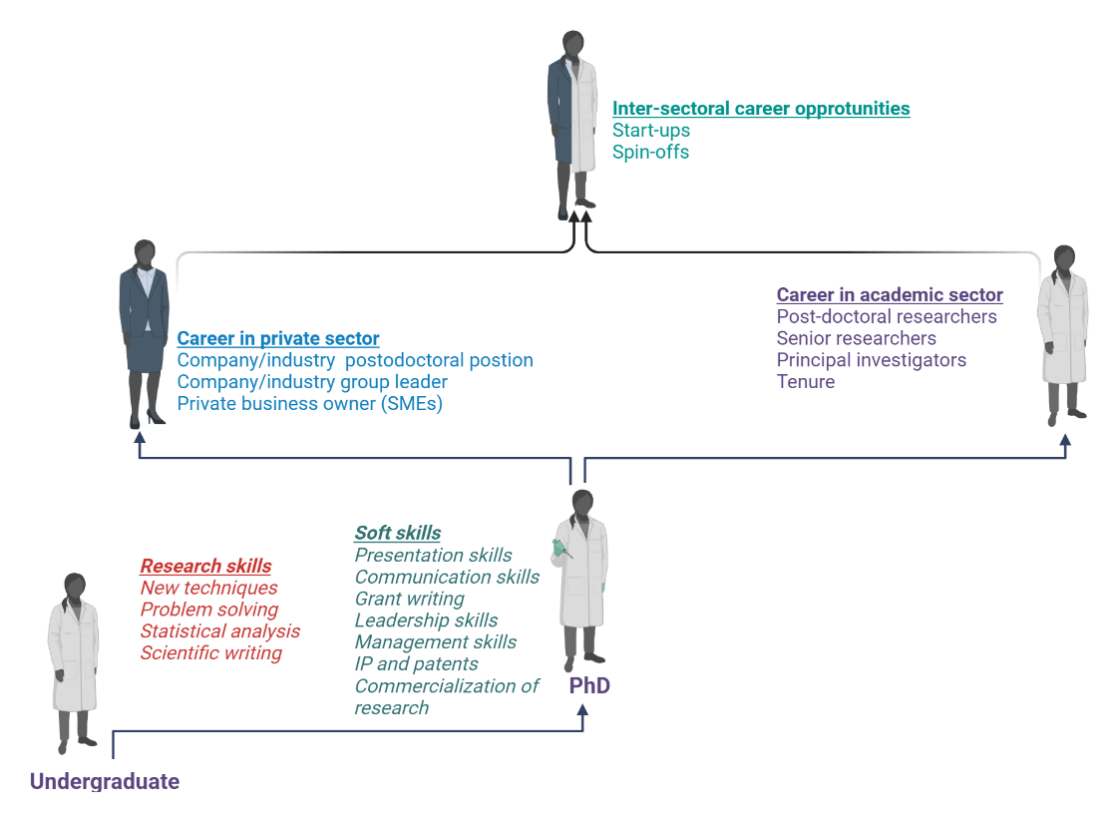
Opportunities for carrier development of ECRs. Figure created in BioRender.

According to Denton *et al.,* around 80 percent of US ECRs (postdocs) in life sciences are employed in academic sector, whilst the rest are working in governmental institutions, industry, or non-profit organizations ^56^. However, the opportunities for researchers to leave academia and work in the private sector as employees or entrepreneurs have significantly increased in the past decade. Large funds are being designated for the development of technology and innovation parks that are seen as crucial ecosystems where innovations can thrive ^57^. The scientific achievements produced in universities are being exploited through creation of spin-off or start-up companies. This, in fact, became one of the most widespread approaches to commercializing scientific discoveries ^58^. Thus, scholars nowadays should gain experience in the private sector through internship programs, and learn about the commercialization of research, technology transfer steps, technology readiness levels, intellectual property, and patents ( Figure 8). By doing so, academics can bridge the gaps between the academic and non-academic sectors, and open doors to new career opportunities.

## Obstacles in acquiring soft skills

The standards and competition in today’s research environment are continuously increasing. Even though the aforementioned soft skills significantly contribute to the advancement of an ECR’s career, they might not be sufficient for career progress due to various external factors. Structural disparities, such as, ethnicity, gender, disabilities, health conditions, and institutional barriers, can notably impede an ECR's acquisition of transferable skills during their research journey ^59^. Women and other marginalized gender identities, people with disability and minority ethnic groups experience with numerous disadvantages. Gender disparities in research can rise from implicit biases and added need for work-life balance among women, resulting in fewer opportunities. ECRs dealing with disabilities or chronic health conditions may face challenges in managing their workloads and may encounter difficulties in accessing the necessary resources and support to develop these transferable skills. For instance, people with disabilities are 30 percent less likely to enter professional roles compared to those without disabilities ^60^. Additionally, students from low-income countries traveling to explore an international and developed research environment may face problems due to financial constraints, language barriers, cultural differences and visa restrictions that hinder their ability to pursue an advanced research career. These inequalities significantly affect ECRs' productivity during their professional development and their ability to establish research networks.

Therefore, besides supporting emerging scholars in gaining transferable skills, it is imperative to firstly recognize and then address the structural imbalances some ECRs face. This can be partially achieved by researchers themselves supporting peers who face disadvantages. Additionally, established scientists can make a significant difference by providing development opportunities and overseeing research practices at the institutional level. Creating an inclusive and welcoming environment while providing mentorship and offering options of professional development can help students from marginalized groups to overcome these obstacles and thrive in their careers.

## Conclusions and key messages

The life of an ECR is made of successes and failures. Even though sometimes failures can be more frequent than successes, the most important thing to remember is to keep going and to not feel discouraged. Soft skills and the helpful suggestions provided in this article should help ECRs as well as other more advanced researchers to build their niche in the highly competitive research arena. Writing a scientific publication is an essential step in a researcher’s life and sometimes it takes several rounds of revision before a paper is accepted. However, for sure these revisions will highly improve the quality of the paper. Grant applications take an increasing amount of time as long as the ECR becomes an established researcher. Even though most prestigious grant schemes have very low success rates, close to 10 percent or below in some cases, never give up! Only the most hard-worker and galvanized applicants will succeed and will be rewarded by the satisfaction of having won a high-level competition. Keep the eyes widely open, share ideas and findings with colleagues, talk to peoples with complementary expertise, be part of research networks, be proactive, multitask, and forget about your shyness. These are key soft skills that will help ECRs achieve a successful and enjoyable research career.

## Data Availability

No data are associated with this article.
