# Supplementary material for: The science behind soft skills: Do’s and Don’ts for early career researchers and beyond. A review paper from the EU-CardioRNA COST Action CA17129
Source: Open Res Eur. 2023 Oct 12;3:55. Originally published 2023 Apr 13. [Version 2] doi: 10.12688/openreseurope.15746.2 (PMC11058455; doi:10.12688/openreseurope.15746.2)
Supplement: Peer review history

# Reviewer report: Reviewer response for version 2

**DOI:** 10.21956/openreseurope.18074.r39632

**Published:** 2024-04-29

**Authors:** Monica Pereira

**Affiliations:** 1 Brunel University London, London, England, UK

Thank you for requesting me to review this manuscript. I can report that this manuscript is well written and very well structured which makes it easy to read and understand. I can see this making a significant contribution to the academic community as it highlights key issues and good recommendations for soft skill demonstration in ECRs. The authors put forward many aspects of ECR development and training. Considering that I am joining for a second review line, I do not have many comments as the paper speaks for itself.

 In the paper, there is some mention of obstacles of acquiring soft skills in various disabilities, genders etc. It would be good to be more specific on the matter with some suggestions for these populations too. Considering that as an academic community we are actively trying to support these groups in their career development. I propose that there could be a whole new section around inclusivity, diversity and culture in the workplace which would detail how to be an ally in the workplace and contribute to workplace environment that everyone feels safe to work in. It could also detail how to showcase this in the interview stage by demonstrating this through a number of personal experiences and empathy. This would be inline with leadership skills listed in Figure 1.

https://commission.europa.eu/about-european-commission/organisational-structure/people-first-modernising-european-commission/people-first-diversity-and-inclusion_en

Where applicable, are recommendations and next steps explained clearly for others to follow? (Please consider whether others in the research community would be able to implement guidelines or recommendations and/or constructively engage in the debate)

Yes

Does the article adequately reference differing views and opinions?

Yes

Are all factual statements correct, and are statements and arguments made adequately supported by citations?

Yes

Is the rationale for the Open Letter provided in sufficient detail? (Please consider whether existing challenges in the field are outlined clearly and whether the purpose of the letter is explained)

Yes

Is the Open Letter written in accessible language? (Please consider whether all subject-specific terms, concepts and abbreviations are explained)

Yes

Reviewer Expertise:

Soft skills between higher education and industry, business psychologist consultant and Director of Employability in my Department.

I confirm that I have read this submission and believe that I have an appropriate level of expertise to confirm that it is of an acceptable scientific standard, however I have significant reservations, as outlined above.

# Reviewer report: Reviewer response for version 1

**DOI:** 10.21956/openreseurope.17010.r34898

**Published:** 2023-10-06

**Authors:** Marialuisa Villani

**Affiliations:** 1 Department of Political and Social Sciences, Universita degli Studi di Bologna, Bologna, Emilia-Romagna, Italy

The paper provides an extensive exploration of topics related to the career development of Early Career Researchers (ECRs) and how soft skills can contribute to their professional growth. The authors offer comprehensive insights into various research-related tasks, such as conducting literature reviews and following research protocols in the science field.

 However, the paper predominantly portrays the transfer of soft skills as a linear process and briefly acknowledges the challenges that the educational environment may pose. While they acknowledge the importance of a strong trainer-trainee relationship at the outset, they do not delve into potential critical aspects of skill transfer.

 In the open letter, the authors imply a linear progression from the acquisition of soft skills to the success of ECRs. Nevertheless, they fail to consider factors such as gender, disabilities, health, and nationality, which can significantly impact the acquisition of transferable skills during PhD or Post-Doc programs. These inequalities also affect ECRs' productivity during their career development and their ability to establish research networks. The paper only briefly touches on the influence of available funding on networking and the open science publication process.

 As it stands, the paper places the onus of ECR success or underperformance squarely on the individual trainee. However, within sociological literature, including education and work studies (e.g., Bourdieu 1984, Bozzon et al. 2018, Crew T.), scholars have emphasized the role of structural inequalities related to gender, disabilities, ethnic origin, and health conditions.

 While I appreciate the recommendations put forth by the EUCardioRNA COST Action CA17129, if they aim to provide meaningful and practical guidance for students seeking to pursue a Ph.D. program and a research career, it is essential to comprehensively address all aspects of an academic career path. This should encompass not only recognizing the importance of acquiring soft skills but also acknowledging the influence of structural inequalities and their impact on the journey of ECRs.

Where applicable, are recommendations and next steps explained clearly for others to follow? (Please consider whether others in the research community would be able to implement guidelines or recommendations and/or constructively engage in the debate)

Yes

Does the article adequately reference differing views and opinions?

Partly

Are all factual statements correct, and are statements and arguments made adequately supported by citations?

Partly

Is the rationale for the Open Letter provided in sufficient detail? (Please consider whether existing challenges in the field are outlined clearly and whether the purpose of the letter is explained)

Yes

Is the Open Letter written in accessible language? (Please consider whether all subject-specific terms, concepts and abbreviations are explained)

Yes

Reviewer Expertise:

Sociology of Education, Education Policies, Higher Education Studies

I confirm that I have read this submission and believe that I have an appropriate level of expertise to confirm that it is of an acceptable scientific standard, however I have significant reservations, as outlined above.

## References

[1] : Homo academicus. *Paris, Les Éd. de Minuit.* .1984;

[2] : Gender and precarious careers in academia and research: Macro, meso and micro perspectives, In A. Murgia, B. Poggio (eds) Gender and Precarious Research Careers: A Comparative Analysis. *London-New York, Routledge* .2018;15-49

[3] : Precarity, Higher Education and Working-Class Academics. *Cham, Palgrave Pivot* .2020;45-67

# Response

**Published:** 2023-11-17

**Authors:** Yvan Devaux

**Affiliations:** Luxembourg Institute of Health, Luxembourg

***Reviewer 2 comments*** *:* *The paper provides an extensive exploration of topics related to the career development of Early Career Researchers (ECRs) and how soft skills can contribute to their professional growth. The authors offer comprehensive insights into various research-related tasks, such as conducting literature reviews and following research protocols in the science field.* *However, the paper predominantly portrays the transfer of soft skills as a linear process and briefly acknowledges the challenges that the educational environment may pose. While they acknowledge the importance of a strong trainer-trainee relationship at the outset, they do not delve into potential critical aspects of skills transfer.* *In the open letter, the authors imply a linear progression from the acquisition of soft skills to the success of ECRs. Nevertheless, they fail to consider factors such as gender, disabilities, health, and nationality, which can significantly impact the acquisition of transferable skills during PhD or Post-Doc programs. These inequalities also affect ECRs' productivity during their career development and their ability to establish research networks. The paper only briefly touches on the influence of available funding on networking and the open science publication process.* *As it stands, the paper places the onus of ECR success or underperformance squarely on the individual trainee. However, within sociological literature, including education and work studies (e.g., Bourdieu 1984, Bozzon et al. 2018, Crew T.), scholars have emphasized the role of structural inequalities related to gender, disabilities, ethnic origin, and health conditions.* *While I appreciate the recommendations put forth by the EUCardioRNA COST Action CA17129, if they aim to provide meaningful and practical guidance for students seeking to pursue a Ph.D. program and a research career, it is essential to comprehensively address all aspects of an academic career path. This should encompass not only recognizing the importance of acquiring soft skills but also acknowledging the influence of structural inequalities and their impact on the journey of ECRs.*

Answer: We thank this reviewer for her comments and fully agree with her recommendations. We have addressed the raised points in the updated version of the manuscript by including a chapter as follows.  “ **Obstacles in acquiring soft skills ** The standards and competition in today’s research environment are continuously increasing. Even though the aforementioned soft skills significantly contribute to the advancement of an ECR’s career, they might not be sufficient for career progress due to various external factors. Structural disparities, such as, ethnicity, gender, disabilities, health conditions, and institutional barriers, can notably impede an ECR's acquisition of transferable skills during their research journey ^59^. Women and other marginalized gender identities, people with disability and minority ethnic groups experience with numerous disadvantages. Gender disparities in research can rise from implicit biases and added need for work-life balance among women, resulting in fewer opportunities. ECRs dealing with disabilities or chronic health conditions may face challenges in managing their workloads and may encounter difficulties in accessing the necessary resources and support to develop these transferable skills. For instance, people with disabilities are 30 percent less likely to enter professional roles compared to those without disabilities ^60^. Additionally, students from low-income countries traveling to explore an international and developed research environment may face problems due to financial constraints, language barriers, cultural differences and visa restrictions that hinder their ability to pursue an advanced research career. These inequalities significantly affect ECRs' productivity during their professional development and their ability to establish research networks. Therefore, besides supporting emerging scholars in gaining transferable skills, it is imperative to firstly recognize and then address the structural imbalances some ECRs face. This can be partially achieved by researchers themselves supporting peers who face disadvantages. Additionally, established scientists can make a significant difference by providing development opportunities and overseeing research practices at the institutional level. Creating an inclusive and welcoming environment while providing mentorship and offering options of professional development can help students from marginalized groups to overcome these obstacles and thrive in their careers.”

# Reviewer report: Reviewer response for version 1

**DOI:** 10.21956/openreseurope.17010.r31146

**Published:** 2023-07-18

**Authors:** Sandra Alexandra Catherine Buttigieg

**Affiliations:** 1 Health Systems Management and Leadership, Faculty of Health Sciences, University of Malta, Msida, Malta; 2 Clinical Performance Unit, Patient Safety and Quality Improvement, Mater Dei Hospital, Buenos Aires, Autonomous City of Buenos Aires, Argentina

Thank you for asking me to review this manuscript - which is extremely well-written, structured and illustrated. It is indeed a report that is useful for ECRs. I would like to mention several points which the authors may find useful to improve their manuscript. This is only meant to be constructive criticism.

1. Leadership should be in the list of soft skills in Figure 1. it is mentioned sparingly, albeit illustrated in Figure 2 together with adaptability as transferable skills. An ECS should have the vision in terms of the strategic positioning/purpose of research so as to achieve maximum impact and implementation. The pandemic has shown us the importance of compassionate, agile, adaptive and distributed/collaborative leadership. Leadership is an important process that could make or break a team of researchers. There is ample research on destructive toxic leadership in particular in highly competitive environments.
2. Systems thinking: as part of patient safety and risk management, ECRs should be able to understand the principles of systems thinking that helps in identifying challenges with impact, implementation, and adoption of innovation. 
3. In line with UN's SDGs, a great deal of attention is being given to both digital and green skills that ensure sustainability of health systems. In particular, the importance of circular economy and its 3R's reduce, reuse and recycle should be important skills to acquire.

Where applicable, are recommendations and next steps explained clearly for others to follow? (Please consider whether others in the research community would be able to implement guidelines or recommendations and/or constructively engage in the debate)

Yes

Does the article adequately reference differing views and opinions?

Yes

Are all factual statements correct, and are statements and arguments made adequately supported by citations?

Yes

Is the rationale for the Open Letter provided in sufficient detail? (Please consider whether existing challenges in the field are outlined clearly and whether the purpose of the letter is explained)

Yes

Is the Open Letter written in accessible language? (Please consider whether all subject-specific terms, concepts and abbreviations are explained)

Yes

Reviewer Expertise:

Clinical performance and quality assurance in healthcare; Patient safety and clinical risk management; Organisational behavior, SHRM, SHRD; Research methods; Operations management/research (length of stay, readmissions, delayed discharges); Health policy issues (ageism, migrant health, One Health); Information technology in health care management

I confirm that I have read this submission and believe that I have an appropriate level of expertise to confirm that it is of an acceptable scientific standard.

# Response

**Published:** 2023-11-17

**Authors:** Yvan Devaux

**Affiliations:** Luxembourg Institute of Health, Luxembourg

***Reviewer 1 comments:*** *Thank you for asking me to review this manuscript - which is extremely well-written, structured and illustrated. It is indeed a report that is useful for ECRs. I would like to mention several points which the authors may find useful to improve their manuscript. This is only meant to be constructive criticism.*

1. *Leadership should be in the list of soft skills in Figure 1. it is mentioned sparingly, albeit illustrated in Figure 2 together with adaptability as transferable skills. An ECS should have the vision in terms of the strategic positioning/purpose of research so as to achieve maximum impact and implementation. The pandemic has shown us the importance of compassionate, agile, adaptive and distributed/collaborative leadership. Leadership is an important process that could make or break a team of researchers. There is ample research on destructive toxic leadership in particular in highly competitive environments.*

Reply- We thank the reviewer for the comment. Leadership has been added in the list of soft skills in Figure 1.

1. *Systems thinking: as part of patient safety and risk management, ECRs should be able to understand the principles of systems thinking that helps in identifying challenges with impact, implementation, and adoption of innovation. *

Reply- We thank the reviewer for this important recommendation. We have included the concept of systems thinking in the transferable skills section. “Intertwined with critical thinking is systems thinking, which involves analyzing complex problems by considering how the different parts of the problem are interconnected and interdependent on each other. By considering all aspects of the problem, systems thinking helps to identify the challenges of implementing and using the innovation.”

1. *In line with UN's SDGs, a great deal of attention is being given to both digital and green skills that ensure sustainability of health systems. In particular, the importance of circular economy and its 3R's reduce, reuse and recycle should be important skills to acquire.*

Reply- Thank you for the insights. We have included this in the good scientific practices section, supporting the principles of 3R and ARRIVE guidelines. “In this regard, the United Nations has proposed Sustainable Development Goals (SDGs; https://sdgs.un.org/goals). Following the SDGs, it is essential for the researchers to focus on both digital and green skills which could help in the use of technology to manage and solve problems and to reduce the environmental impact of research activities ensuring the sustainability of health systems.”
